# FUTURE PLANNING AMONG OLDER CAREGIVERS OF FAMILY MEMBERS WITH INTELLECTUAL DISABILITY OR MENTAL ILLNESS

**DOI:** 10.1093/geroni/igz038.1060

**Published:** 2019-11-08

**Authors:** Fei Wang, Yangdi Han

**Affiliations:** 1 Jack, Joseph and Morton Mandel School of Applied Social Sciences, Case Western Reserve University, Cleveland, Ohio, United States; 2 School of Social Development and Public Policy, Fudan University, Shanghai, Shanghai, China

## Abstract

Objectives: This study aims to examine future planning among older caregivers for family members with intellectual disability or mental illness, focusing on preferences, predictors and barriers. Method: Data were drawn from 260 caregivers (aged 50 or older) to a family member with intellectual disability or mental illness in Shanghai, China. Caregivers rated six types of future care arrangement under three circumstances: (1) the ideal situation, (2) unable to provide care due to age-related illnesses, and (3) caregivers are deceased. Socio-demographic factors associated with future planning were examined using multinomial logistic regression. Caregivers also rated twelve barriers to future planning. Results: Government-subsidized care facility is the most preferable care arrangement across the three circumstances. While continuing family care was still preferred if caregivers were to become sick or deceased, it was a less preferred option in the ideal situation. Common barriers were the cost of institutional care and the inadequate skills of the staff. Regarding the predictors of future planning, the older the caregivers were, the less likely they had no future plans. Caregivers were more likely to prefer family care over institutional care if their family members had mild impairment. Caregivers of a family member with mental illness were more likely to have no future planning than caregivers of a family member with intellectual disability. Conclusion: This study identified the needs of older caregivers for future planning specific to different circumstances. It also identified demographic profiles of future planning and the caregiver population at risk of no future planning.

